# Effectiveness of box trainers in laparoscopic training

**DOI:** 10.4103/0972-9941.33274

**Published:** 2007

**Authors:** Anender Kaur Dhariwal, Ramkrishna Y Prabhu, Abhay N Dalvi, Avinash N Supe

**Affiliations:** Department of Surgical Gastroenterology, Seth GS Medical College and KEM Hospital, Parel, Mumbai - 400 012, India

**Keywords:** Box-trainers, endotraining, skill assessment

## Abstract

**Rationale and Objectives:**

Various devices are used to aid in the education of laparoscopic skills ranging from simple box trainers to sophisticated virtual reality trainers. Virtual reality system is an advanced and effective training method, however it is yet to be adopted in India due to its cost and the advanced technology required for it. Therefore, box trainers are being used to train laparoscopic skills. Hence this study was undertaken to assess the overall effectiveness of the box-training course.

**Study Procedure:**

The study was conducted during six-day laparoscopic skills training workshops held during 2006. Twenty five surgeons; age range of 26 to 45 years, of either sex, who had not performed laparoscopic surgery before; attending the workshop were evaluated. Each participant was given a list of tasks to perform before beginning the box-training course on day one and was evaluated quantitatively by rating the successful completion of each test. Evaluation began when the subject placed the first tool into the cannula and ended with task completion. Two evaluation methods used to score the subject, including a global rating scale and a task-specific checklist. After the subject completed all sessions of the workshop, they were asked to perform the same tasks and were evaluated in the same manner. For each task completed by the subjects, the difference in the scores between the second and first runs were calculated and interpreted as an improvement as a percentage of the initial score.

**Statistical Analysis:**

Wilcoxon matched-paired signed-ranks test was applied to find out the statistical significance of the results obtained.

**Results:**

The mean percentage improvement in scores for both the tasks, using global rating scale, was 44.5% ± 6.930 (Mean ± SD). For task 1, using the global rating scale mean percentage improvement was 49.4% ± 7.948 (Mean ± SD). For task 2, mean percentage improvement using global rating scale was 39.6% ± 10.4 (Mean ± SD). Using Wilcoxon matched-paired signed-ranks test, 2-tailed *P*-value<0.0001 which is extremely significant.

**Conclusion:**

This study confirms that a short-term, intensive, focused course does improve laparoscopic skills of trainees. Box-trainers can be used to change the present day didactic training into objective and competency-based. Global rating scale and checklist provide an inexpensive and effective way of objective assessment of performance of laparoscopic skills.

## INTRODUCTION

Recognizing the growing role of laparoscopy in modern surgery, residency programs have rapidly incorporated it into their training regimen. Various devices ranging from simple box trainers, animal models to sophisticated virtual reality trainers[1–4] are used to aid in this education of laparoscopic skills. Virtual reality system is an advanced and effective training method, however it is yet to be adopted in India due to its cost and the advanced technology required for it.[5] Therefore, box trainers are being used to train laparoscopic skills.[6] The question that is often raised, by both surgeons and the public, is whether these training sessions are effective in actually improving one's skills enough to become proficient at performing laparoscopic surgery[3,7,8] In this paper, we explore an inexpensive and easy method of objective evaluation and test it on surgeons who attend a training workshop. The aim of this paper is to assess improvement in dexterity of surgical trainees and overall effectiveness of the box-training exercises.

## MATERIALS AND METHODS

The study was carried out during six-day laparoscopic training workshops in the Department of Surgical Gastroenterology of Seth G.S. Medical College and KEM hospital in year 2006. Permission of the Institutional Ethics Committee of Seth GS Medical College and KEM Hospital, Mumbai was taken before the commencement of the study.

All surgeons within the age range of 26 to 45 years, of either sex (male 19, female 6), who had no prior laparoscopic experience or were not exposed to laparoscopy were included in the study after due consent. Surgeons had not personally performed or assisted or handled the equipment or instruments prior to attending this course.

### Study procedure

#### Set-Up:

The department of Surgical Gastroenterology, K.E.M. Hospital has an Endo-lab that consists of an isolated room with box-trainers. Laparoscopic training course is a six-day course. Each day there are lectures, operative demonstrations and a three hour training session on the box-trainer that included one hour of supervised performance and two hours of practice (The two hours of practice time also includes the time when one candidate held the camera for the other person to get the feel of how a camera person is Important in laparoscopic surgery as he/she is eyes of the surgeon and success of a procedure is a team effort). Participants perform six tasks on six consecutive days in the following order: day 1- bead transfer; day 2- transferring the sugar cubes; day 3- transferring the rings from one pin to another; day 4-bowel tracing; day 5- pattern cutting; day 6- suture drill. They were given two hours to practice each task.

#### Box-Trainer:

**Figure 1:**
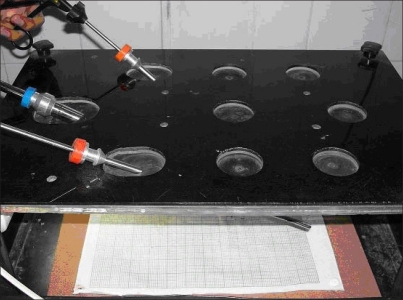
Box trainer developed to simulate endoscopic procedures

**Figure 2:**
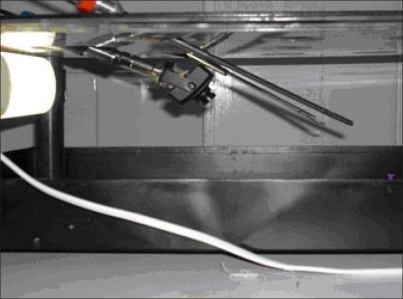
Box trainer with the endoscope and instruments

Locally manufactured box-trainer contains a board placed in a black training box fitted with rubber gaskets to accommodate cannulae for the scope and tools [Figures 1 and 2]. A fiber-optic light source and camera equipment is used and the image is displayed on a video monitor. The following instruments are used 1. Atraumatic grasping forceps, both jaws opening 2. Grasping/dissecting forceps curved left, both jaws opening (Maryland Dissector) 3. Scissors curved left, both blades opening (Metzenbaum) 4. Modular needle holder, straight with carbide insert with top lock

### Pre-training evaluation

Each participant was given two tasks; one from basic-skills and other from advanced-skills to perform. The evaluation was done by a senior faculty member and a trained observer.

#### Task 1. Bead transfer:

**Figure 3:**
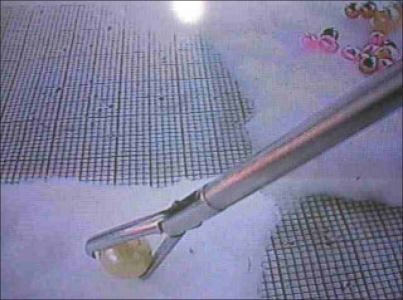
Transferring the bead (task 1)

The task consisted of picking up and transferring first the pink, then yellow and then white bead one by one; from one cotton pad to another and arranging then in a straight line [Figure 3].

#### Task 2. Suture drill:

**Figure 4:**
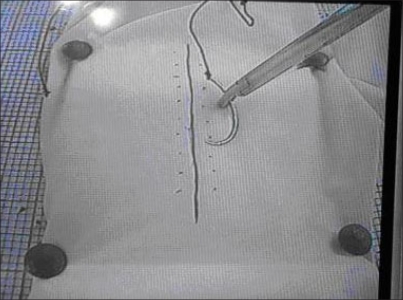
Suture drill (task 2)

The task consisted of repairing the incision on the glove with a single suture using 3-0 silk sutures on a curved tapered needle and to make an intra-corporeal knot and secure each knot. Attention was paid to the basic skills involved in suturing like tracing, needle holding, taking proper bites, tying knot and cutting off the extra suture to a short length [Figure 4].

Each participant was asked to perform these tasks before beginning the training course on day one. They were evaluated quantitatively by rating the successful completion of each test. Evaluation began when the subject placed the first tool into the cannula and ended when the task was complete. Two evaluation methods were used to score the participants, including a global rating scale[9,10] (maximum score =20) [Table 1] and a task-specific checklist[9] (maximum score=13) [Table 2]. A global rating scale was recorded for each task the participant performed and checklist for the suture drill.

**Table 1:** Global rating scale

Rating					

Variable	1	2	3	4	5
Time and motion moves	Many unnecessary		Efficient time and motion, but some unnecessary moves		Economy of movement and maximum efficiency
Instrument handling	Repeatedly makes tentative or awkward moves with instruments		Competent use of instruments, although occasionally appeared stiff or awkward		Fluid moves with instruments and no awkwardness
Knowledge of instruments	Frequently asked for the wrong instrument or used an inappropriate instrument		Knew the names of most instruments and used appropriate instrument for the task		Obviously familiar with the instruments required and their names
Use of assistants	Consistently placed assistants poorly or failed to use assistants		Good use of assistants most of the time		Strategically used assistant to the best advantage at all times

**Table 2:** Task based checklist - Suturing on glove: Interrupted end-to-end suturing on gloves. Score one point for each correctly performed action

Procedural step	Correctly performed	Incorrectly performed
Selects appropriate instruments (non-tooth forceps)		
Needle oriented-no twisting, correct stay placement		
Correct needle holding technique		
Needle driver stabilized with good hand position		
Needle enters glove at right angles
Single attempt at passage through the glove		
Follow through on curve of needle on entrance		
Follow through on curve of needle on exit		
Adequate bites taken (>3 mm from edge of glove)		
Use of forceps to handle needle		
Square knots		
Knots placed to one side of suture line		
Suture cut to appropriate length		

### Post-training evaluation

After the participants completed all sessions of the workshop, they were asked to perform the same tasks and were evaluated in the same manner. For each task completed by the subjects, Wilcoxon matched-paired signed-ranks test was used to find the statistical significance between the pretraining and posttraining scores.

### Statistical analysis

For each task completed by the subjects, Wilcoxon matched-paired signed-ranks test was used to find the statistical significance between the pre training and post training scores and a *P* value <0.05 was considered to statistically significant. Difference in scores between the second and first runs was calculated and interpreted as an improvement as a percentage of the initial scores.

## RESULTS

All twenty-five surgeons completed the study. The mean percentage improvements in scores by global rating scale stratified by task are listed in Table 3. (Maximum score = 20). The mean percentage improvement in the scores using global rating scale was 44.5% ± 6.930 (Mean ± SD).

**Table 3:** Mean percentage improvement in scores (using global rating scale) by task

	Mean Pre-training scores	Mean Post-training scores	Percentage Improvement	

			Mean	S
Task 1	37	87	49.4	7.984
Task 2	26	65.6	39.6	10.4
Total	31.5	76.3	44.5	6.930

Value in parentheses are percentages, SD: Standard deviation

For task 1 [Table 4], using the global rating scale mean percentage improvement was 49.4% (Pre 7.4 ± 1.97 vs. Post 17.4 ± 1.41) with a standard deviation of 7.948. Using Wilcoxon matched-paired signed-ranks test, 2-tailed *P*-value <0.0001 which is extremely significant and Non-parametric spearman correlation coefficient is 0.6363.

**Table 4:** Task 1 (bead transfer): Pre and post training scores (out of 20) using global rating scale

	Pre-training score	Post-training score
Mean	7.4	17.4
Standard deviation	1.979	1.414
Median	8	17
Inter-quartile range	4	2

Using Wilcoxon matched-paired signed-ranks test, 2-tailed *P*-value<0.0001 which is extremely significant.

For task 2 [Table 5], mean percentage improvement using global rating scale was 39.6% (Pre 5.2 ± 1.04 vs. Post 13.12 ± 1.96) with a standard deviation of 10.4. Using Wilcoxon matched-paired signed-ranks test, 2-tailed *P*-value <0.0001 which is extremely significant and Non-parametric spearman correlation coefficient is 0.1797.

**Table 5:** Task 2 (suture drill): Pre and post training scores (out of 20) using global rating scale

	Pre-training score	Post-training score
Mean	5.2	13.12
Standard deviation	1.041	1.965
Median	5	13
Inter-quartile range	2	2

Using Wilcoxon matched-paired signed-ranks test, 2-tailed *P*-value<0.0001 which is extremely significant.

The mean improvement in suturing skills [Table 6] using task-based checklist was 45.84% with a standard deviation of 15.77 (Pre 3.52 ± 1.61 vs. Post 9.0 ± 2.08). Using Wilcoxon matched-paired signed-ranks test, 2-tailed *P*-value <0.0001 which is extremely significant and Non-parametric spearman correlation coefficient is 0.278.

**Table 6:** Task 2 (suture drill): Pre and post training scores (out of 13) using checklist

	Pre-training score	Post-training score
Mean	3.52	9.
Standard deviation	1.661	2.084
Median	3	8.5
Inter-quartile range	1.5	2.5

Using Wilcoxon matched-paired signed-ranks test, 2-tailed *P*-value<0.0001 which is extremely significant.

## DISCUSSION

In the mid to late 1980s, the advent and widespread demand for laparoscopic cholecystectomy disrupted the fundamentals of traditional surgical training. It was not only subjects who needed training but now also practicing surgeons who did not have the luxury of time to acquire these new and deceptively difficult skills. This MIS revolution eventually led surgeons to rethink how best to train surgical skills.

One approach to skills acquisition was use of simulation, proposed by Satava[11] in the early 1990s. Over the past decade evidence has progressively accumulated in favor of use of simulation as a means of training and as an evaluation tool in the field of laparoscopy. Before it is incorporated in the already hectic regular curriculum, training and evaluation methodology needs to be standardized, internal validity established and usefulness of such training proven beyond doubt.

While surgical skills simulators are being produced in ever-increasing numbers, there is still confusion about how to use simulators to teach surgical skills. The underlying assumption seems to be that individuals, who have performed the required number of procedures will be a safe practitioner. A fundamental flaw with this approach is that it ignores individual variability with respect to skill acquisition. Setting a fixed number of procedures or number of training hours is not an optimal approach to learning. Thus the various methods of surgical skills training till now were so much knowledge-oriented and subjective. So there is a need to make training objective and competency-based.

In this study, the subjects were made to perform two tasks [one basic skill (bead transfer) and one advanced skill (suture drill)]. Bead transfer was designed to emphasize basic skills like instrument handling, use of assistant, getting orientation to the two-dimensional images on the video screen and work with proper hand-eye co-ordination. Since the participants had not performed laparoscopic surgery before they demonstrated extremely significant improvement in this basic task. The post-training scores for this task were between 75%-100% for almost all the participants. This can be attributed to the training.

Suture drill, the advanced and specific skill that was tested was the ability to place a suture and make an intra-corporeal knot. Though using sutures to approximate tissues in laparoscopic procedures can be very difficult and time-consuming, surgeons should always be prepared for the unexpected need to use sutures in situations where clips fail. In addition as the application of laparoscopy expands to include more complex procedures, suturing and knot tying on a video screen will be an important part of surgery in the future.[12] In suture drill task, the subjects were asked to place a single stitch on an incision on a glove using 3-0 silk suture on a curved tapered needle. Attention was paid to the basic skills involved in suturing like tracing, needle holding, taking proper bites, tying knot and cut off the extra suture to a short length. They formed a standard surgeon's knot intracorporeally. The participants demonstrated extremely significant improvement in suturing and tying, also evident from the percentage improvement in the global rating scale and checklist scores for suturing. However the posttraining scores for suturing were not as high as those for bead transfer. This may be because suturing is a difficult task and three hours of practice on the box trainer may not be enough to bring the scores to 75% to 100%.

By using both, global rating scale and task specific checklist for assessment, Regehr *et al.*[9,13] have shown that checklists do not add any additional value to the assessment process and that their reliability is lower than that for the global rating scale.[14,15] We have found both the scales useful. The most ideal method of gaining real operative experience outside the operating room would be practicing complete procedures like laparoscopic cholecystectomy or Nissen fundoplication on live animals such as the domestic swine,[14] but this is costly and requires an experienced staff of an anesthetist, a veterinarian and a lab. technician. The advantage of box-training for objective evaluation of basic skills is twofold. First the scenario for testing is easily reproducible. The performance is not biased by the variations in anatomy or physiologic response found in animals. The exact same test can be repeated in identical fashion at any location at any time. Second the equipment is inexpensive, reusable and easy to set up quickly without an experienced staff.

Munz and colleagues[16] did a study to compare the virtual reality simulator with the classical box trainer and determine whether one has advantage over the other. Twenty-four novices were tested to determine their baseline laparoscopic skills and then randomized into the following three groups: Lap Sim, box trainer and no training (control). After three weekly training sessions lasting 30 minutes each, all subjects were reassessed. Assessment included motion analysis and error scores. Nonparametric tests were applied and *P*<0.05 was deemed significant. Both trained groups made significant improvements in all parameters measured (*P*<0.05). Compared to the controls, the box trainer group performed significantly better on most of the parameters, whereas the LapSim group performed significantly better on some parameters. There were no significant differences between the LapSim and box trainer groups

Scott *et al.*[15] have shown that inexperienced subjects (undergraduate students) benefit more from training on the simulators as compared to the more experienced post graduate students. Maximum benefit could be achieved with 30-35 repetitions on such tasks. In addition, Powers *et al.*[17] demonstrated that among participants with similar experience, those with lower initial scores seem to benefit more from tasks involving basic skills. Furthermore, the final scores achieved after two weeks of training were similar for groups starting with low and higher initial scores.

This study also emphasizes need for objective assessment of laparoscopic skills and use of box trainers to document the same. This will allow training centers to strive to improve the quality of teaching, ensure standardization and change course formats according to the performance of participants. Past experiences such as video watching usually improves only cognitive skills of the participants such as understanding and comprehension of the procedures. To improve psychomotor skills and dexterity, box training or actual performance is essential. Surgeons may have seen the procedures but not personally performed or assisted to understand the difference of two-dimensional vision and haptics. This study proves that short-term; intensive, focused courses do improve laparoscopic skills of surgical trainees. Follow-up studies will be done to assess whether this really improves the clinical skills of surgeons. This would be evaluated by sending a questionnaire to the trainees after one year, enquiring whether it really improved their clinical skills during laparoscopic surgery. The box-trainers can be used to change the present day didactic training into objective and competency-based.

Based on our study, we can conclude that physical laparoscopic simulators certainly have a role as a tool for training and evaluation in the near future. The cost factor, which is particularly relevant in the Indian context, should not be an impediment in the widespread usage of this novel tool. Low-cost substitutes like mirrored-box simulators have been shown to provide a reasonable reflection of relative performance of laparoscopic skills.[18] Thus, the objective of practical and effective laparoscopic skills training and assessment can be realised without the need for expensive equipment.
